# Book Notices

**Published:** 1885-05-20

**Authors:** 


### BOOKS AND PAMPHLETS RECEIVED.

*Mind Reading and Beyond*, by Wm. A. Hovey. Boston: Lee & Sheppard. New York: Charles T. Dillingham.

A work of two hundred pages, cloth, neatly printed, and with many illustratious, designed to show that impressions may be made upon the mind of one man by another. The most successful experiments given are in cases where a large number of persons concentrated their thoughts upon a single purpose, willing the party to do a particular thing, or to see, mentally, a particular object, or to draw upon a blackboard a picture of the object which was impressed upon the mind.

The experiments appear to be fairly given, and, while many failures occur, enough is shown to establish the fact that, under favorable conditions, a passive and susceptible subject *can be impressed* so as to perceive and reproduce the thoughts of another.

*A Practical Treatise on Diseases of Children*, by Eustace Smith, M.D., Fellow of the Royal College of Physicians, Physician to His Majesty the King of the Belgians, Physician to the East London Children's Hospital and to the Victoria Park Hospital for Diseases of the Chest. New York: Wm. Wood & Co., 56 LaFayette Place.

The above work is of very recent issue, is well and neatly published, and contains 844 large octavo pages. It is more full and satisfactory than any work yet issued in the department of Children's diseases.

The opportunities of the author for studying these diseases have been of a superior order, and that through a period of more than twnety years He speaks largely from a clinical standpoint, and is to be the more relied upon. He has not confined himself wholly to the class of diseases which are specially peculiar to childhood, but has included many forms of illness which are capable of being influenced or modified by early age. He claims to have omitted nothing of real value to the practitioner in connection with the diseases of children. We recommend the work as eminently worthy of a place in the library of every physician.

*Notes from the Physiological Laboratory of the University of Pennsylvania*, Edited by N. A. Randolph, M.D., Assistant Demonstrator of Physiology in the University of Pennsylvania; Fellow of the College of Physicians of Philadelphia; Member of the American Philosophical Society of the Assembly of Natural Sciences of Philadelphia, etc., etc., and by Samuel G. Dixon, Assistant Demonstrator of Physiology at the University of Pennsylvania. Printed by J. B. Lippincott & Company: Philadelphia, 1885.

A small work of 88 pages, containing "brief records of facts of interest brought to light in the course of physiological study." The facts disclosed in this little work evince much care and scientific accuracy, and will prove both interesting and useful, especially to the physiologist and the progressive practitioner.

*A Text Book of Practical Medicine*, Designed for the Use of Students and Practitioners of Medicine, by Alfred L. Loomis, M.D., L.L.D., Professor of Pathology and Practical Medicine in the Medical Department of the University of the City of New York, Visiting Physician to Bellevue Hospital, etc., with Two Hundred and Eleven Illustrations. New York: Wm. Wood & Co., 1884.

The above is a large work on practice, containing eleven hundred pages, neatly bound in cloth and printed in plain, excellent type. The author states that the work is "practically a revision and an elaboration of lectures given during the past eighteen years in the Medical Department of the University of New York.

The work, while containing information which must prove of wide and extensive application, is more especially adapted to American physicians. It is practical and well illustrates the practice in this country.

The illustrations are well designed, and add to the interest and usefulness of the work.

